# Assessment of Total Antioxidant Capacity in Serum of Heathy and Stressed Hens

**DOI:** 10.3390/ani10112019

**Published:** 2020-11-03

**Authors:** Stefano Cecchini, Francesco Fazio

**Affiliations:** 1 Department of Sciences, University of Basilicata, Viale dell’Ateneo Lucano 10, 85100 Potenza, Italy; stefano.cecchini@unibas.it; 2 Department of Veterinary Sciences, Polo Universitario Annunziata, University of Messina, 98168 Messina, Italy

**Keywords:** dexamethasone, laying hens, spectrophotometric methods, stress, total antioxidant capacity

## Abstract

**Simple Summary:**

In living organisms, the antioxidant defense system serves to counteract reactive oxygen (ROS) and nitrogen (RNS) species, thereby protecting cellular targets against their oxidative damage; it includes a combination of different substances of endogenous or exogenous origin. Several methods were developed to assess the overall antioxidant capacity or the precise determination of individual key antioxidants. In the present study, the total antioxidant capacity (TAC) in healthy and dexamethasone-stressed hen serum was measured by applying four different spectrophotometric methods intended for both clinical and research studies, which could be automated on clinical auto-analyzers, thus allowing rapid and not expensive data collections. TAC values assessed by all four methods did not change throughout the experimental period in the control group, whereas significant changes were shown by all adopted assays in the stressed group, with some remarkable differences, probably due to the different contribution in each assay of the various antioxidant substances present in the samples. Therefore, when TAC evaluation is necessary to verify if animals are experiencing oxidative stress (OS) or to evaluate possible benefits from an antioxidant-enriched diet, TAC assessment should involve multiple assays, due to the different analytical technologies on which their assessments are based.

**Abstract:**

Total antioxidant capacity (TAC) in healthy and dexamethasone-stressed hens was measured by applying four different spectrophotometric methods—the ferric reducing ability (FRAP) assay, the 2,2′-azino-bis (3-ethylbenzotiazoline-6-sulphonic acid) (ABTS) radical cation decolorization assay, the free radical scavenging activity (FRSA), and the total thiol levels (TTL). TAC assessed by all four methods did not change throughout the experimental period in the control group, whereas significant changes were shown by all adopted assays in the stressed group with some remarkable differences. TAC increased in the stressed group when FRAP and ABTS assays were applied, while it was reduced when sera were assessed by FRSA and TTL assays. Furthermore, FRAP assay was the only test able to show a significant change in TAC immediately after the end of the induced stress. At the end of the experimental period, TAC assessed by ABTS and FRSA assays showed a complete recovery in the stressed group, whereas TAC assessed by FRAP and TTL assays still showed significant persistent differences when compared to the control group. The observed differences in TAC are discussed in the light of the different contribution in each assay of the various antioxidant substances present in the samples.

## 1. Introduction

In living organisms, the antioxidant defense system serves to counteract reactive oxygen (ROS) and nitrogen (RNS) species, thereby protecting cellular targets against their oxidative damage.

The antioxidant system includes a combination of different substances, of endogenous (e.g., antioxidant enzymes, GSH, etc.) or exogenous origin (externally supplied by diets, e.g., vitamin C and E, carotenoids, etc.), which all act together to detoxify oxidative substances to either delay or prevent the oxidative damage of macromolecules [1].

Several methods were developed to assess the overall antioxidant capacity or the precise determination of individual key antioxidants. The methods for the evaluation of the total antioxidant capacity (TAC) are generally based on the content of free radicals scavenged by a test solution, or on the capacity to reduce an oxidized chemical substance [2,3]. One of the main advantages of these tests is that they return the activity of all antioxidants of the biological sample into a single value, thus providing an integrated parameter rather than the simple sum of measurable substances. Thereby, the obtained values are considered as the cumulative effect of all antioxidants of the biological samples. One of the critical points adduced to the application of the assays measuring the cumulative antioxidant potential of biological samples is that the results obtained with different methods are not always comparable, often returning inconsistent data, depending on the different technology used for their assessment [4,5,6]. Therefore, the simultaneous use of different assays represents the pivotal factor for a correct evaluation of the antioxidant status of biological samples.

The application of TAC assays, already in use in human medicine for many years for both clinical and experimental studies, was also adopted to evaluate the effect of oxidative stress (OS) and antioxidant-enriched diets on the livestock welfare and the quality of their derived products [7,8,9,10,11,12,13]. In poultry, in which a combination of several environmental factors often acts in synergy as stressors [1], TAC assays are adopted not only for the evaluation of the blood antioxidant barrier [14,15,16] but also for the evaluation of meat quality, due to its relatively high amount of polyunsaturated fatty acids (PUFA), susceptible to oxidation processes [17,18,19,20]. These tests are rarely used simultaneously [21,22], but in most cases, they are used individually, together with other assays showing single measurable substances, such as antioxidant enzymes [14,16].

Considering the current literature, this study aimed to carry out an extensive comparative analysis of four simple spectrophotometric methods to measure the total antioxidant status in hen serum. The four spectrophotometric assays were the ferric reducing ability (FRAP) assay, the ABTS radical cation decolorization assay, the free radical scavenging activity (FRSA), as DPPH reduction assay, and the total thiol (sulfhydryl group, -SH) levels (TTL). To evaluate the effectiveness of each assay to describe a stressful condition, the chosen tests were comparatively assessed in healthy and stressed hens. Stress was induced by dexamethasone treatment, which is able to cause a reversible and short/medium-term stressful condition, as previously described [12,23]. In particular, we showed that DEX-induced stress has a transient effect on both the specific antibody response and the level of natural antibodies, from which hens recover within two weeks from the conclusion of the induced stress [23].

## 2. Materials and Methods

The experiment was performed at a local farm and all procedures were conducted in strict accordance with European legislation, regarding the protection of animals used for scientific purposes (European Directive 2010/63), as recognized and adopted by the Italian law (DL 2014/26). No animals died during or as a consequence of the conducted experiment.

### 2.1. Experimental Design, Stress Induction and Blood Sampling

Thirty ISA Brown young hens of 16 weeks of age were maintained in floor pens in an environmentally controlled room (20 ± 1 °C), and tagged for identification. Animals were provided with free access to water and feed, using a commercial corn-soybean diet for layers. After 3 weeks of acclimatization, during which the light schedule was progressively modified at 15 h light and 9 h dark, hens were randomly divided into two groups of 15 hens pen^−1^. Hens of the first group were submitted for 6 consecutive days to intramuscular injection of dexamethasone (DEX, Sigma-Aldrich, Milan, Italy), at a dosage of 1.5 mg kg^−1^ body weight, in a final volume of 0.5 mL of sterile saline, to induce a short/medium-term stress condition, as previously described [23]. Here, the DEX-treatment caused transient immune depression. Hens of the second group were injected with 0.5 mL of saline solution for 6 consecutive days and served as controls. At day 0 and before the first DEX-treatment, blood samples were drawn from the brachial vein using a 2.5 mL syringe. Further blood collections were repeated on day-7, -14 and -21, from the beginning of the experiment. Serum was finally obtained after clotting by centrifugation (2500× *g*, 15 min at 4 °C) and stored at −80 °C, until analyzed. To avoid interference due to handling, all procedures necessary for the DEX-treatment and blood collection were carried out by the same operator, as fast as possible.

### 2.2. Analytical Methods

All chemicals used in the analytical methods were purchased from Sigma-Aldrich Company (Milan, Italy).

The ferric reducing ability (FRAP) assay was determined as indicated by Benzie and Strain [24]. The method is based on the principle of the reduction of the ferric-tripyridyltriazine complex to the ferrous form, which causes the development of an intense blue color, which is spectrophotometrically measurable. In brief, 300 mM sodium acetate buffer, pH 3.6, 10 mM tris(2-pyridyl)-s-triazine (TPTZ) in 40 mM HCl and 20 mM iron(III) chloride hexahydrate were mixed in a volume ratio of 10:1:1, to generate FRAP fresh daily prepared solution. Subsequently, 10 µL of samples in duplicates were added to 300 µL FRAP solution in wells of a microtiter plate and the ODs of the reaction mixture were read at 600 nm, after 5 min of incubation at 37 °C in a microplate reader (Model 550, BioRad, Segrate, Milan, Italy). The assay was calibrated with iron (II) sulfate heptahydrate (FeSO_4_·7H_2_O) and the results were expressed in terms of FeSO_4_·7H_2_O equivalents (μM).

The ABTS radical cation decolorization assay was determined as suggested by Re et al. [25]. The method was based on the reduction of the colored 2,2′-azino-bis (3-ethylbenzotiazoline-6-sulphonic acid) (ABTS^•+^) radical cation, to a colorless reduced form, by the antioxidants of samples. The color reduction was then spectrophotometrically measured. In brief, (ABTS^•+^) radical cation was generated by the reaction of 7 mM ABTS with 2.45 mM of potassium persulfate (K_2_S_2_O_8_). The reaction mixture was incubated in the dark for 16 hours at room temperature. Working solutions of ABTS^•+^ were obtained by diluting ABTS^•+^ at the OD of 0.700  ±  0.02 at 734 nm. Subsequently, 300 µL of the ABTS^•+^ solution was added to 3 µL of samples in duplicate in wells of a microtiter plate, and the ODs were read at 660 nm after 6 min of incubation at room temperature in the microplate reader. The assay was calibrated with Trolox and the results are expressed in terms of Trolox equivalents (mM).

The free radical scavenging activity (FRSA) was analyzed by the use of 2,2-Di(4-tert-octylphenyl)-1-picrylhydrazyl (DPPH) reduction assay, based on the reduction of the purple DPPH^•^ to 1,1-diphenyl-2-picryl hydrazine, as described by Blois [26]. In brief, 25 µL of samples were mixed with 475 µL of 10 mM phosphate buffered saline (PBS), pH 7.4, and 500 µL of a 0.1 mM DPPH solution, in absolute methanol. The mixture was kept for 30 min in darkness at ambient temperature, before absorbance reading at 520 nm against blank, using a spectrophotometer (SmartSpec 3000 UV/Vis, Bio-Rad, Segrate, Italy). The absorbance of the sample was compared with that of a reference sample containing only PBS and DPPH solution. The percentage of decrease of DPPH bleeding was calculated by applying the following equation: % of inhibition = [1 − (As/A0)] × 100; where As is the absorbance of sample and A0 is the absorbance of the DPPH solution.

Total thiol (sulfhydryl group, -SH) levels (TTL) were measured as indicated by Hu [27]. Thiols interact with 5,5′-dithiobis-(2-nitrobenzoic acid) (DTNB), and form a highly colored anion with a maximum peak at 412 nm (ε_412_ = 13,600 M^−1^ cm^−1^). In brief, 50 µL of samples were mixed with 1 mL of Tris-EDTA buffer (0.25 M Tris base, 20 mM EDTA, pH 8.2), and the ODs at 412 nm were read against blank in the spectrophotometer. Next, 20 µL of 10 mM DTNB in absolute methanol were added to the solutions. After 15 min at ambient temperature, the ODs were read again against the DTNB blank. TTL were calculated as indicated by Hu [27] and the results were expressed in µM.

Total protein and albumin concentrations were measured by the biuret method using a total protein reagent (T1949; Sigma-Aldrich, Milan, Italy) and by the bromocresol green method [28], respectively, expressing data as bovine serum albumin equivalents (BSA; mg mL^−1^).

### 2.3. Statistical Analysis

Analytical data are presented as mean values ± standard deviation (SD). Data were tested for normal distribution by performing the Shapiro-Wilk test and for variance homogeneity by using Leven’s test. Log transformation was applied when the normality hypothesis was rejected. Two-way analysis of variance (ANOVA) for repeated measures was used to analyze the effect of the independent variables (time and DEX-induced stress) on the obtained analytical data. When a significant overall difference was detected, differences among means were determined by Bonferroni pairwise comparisons. *p*-values less than 0.05 were considered to be statistically significant. All statistical analyses were performed using SigmaPlot Version 11.0 for Windows™ statistical software (Systat Software Inc., San José, CA, USA).

## 3. Results

**Figure 1:**
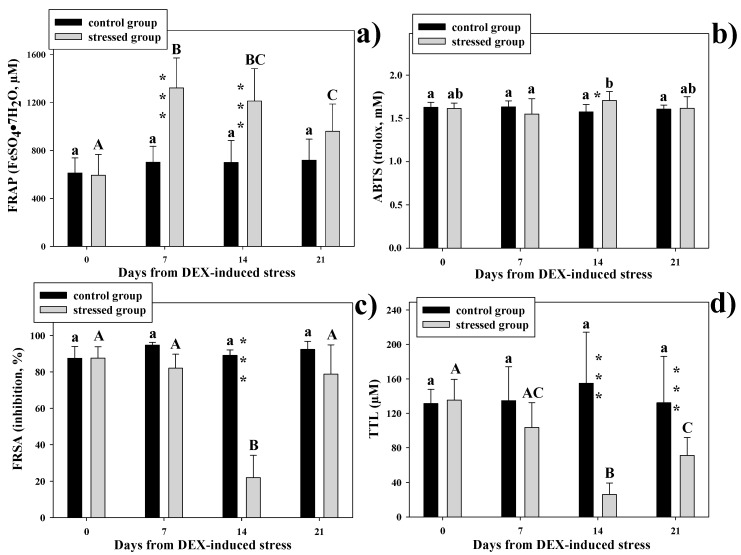
Antioxidant capacity assessed by different methods in healthy and the dexamethasone (DEX)-stressed hens: (**a**): ferric reducing ability (FRAP) assay; (**b**): ABTS decolorization assay; (**c**): free radical scavenging activity (FRSA); and (**d**): total thiol levels (TTL). Values are expressed as means ± standard deviation. Means with asterisks at the same sampling time are statistically different (*, *p* < 0.05; ***, *p* < 0.001). Means of the same group with different letters at different sampling times are statistically different (lowercase letters, *p* < 0.01; capital letters, *p* < 0.001).

Two-way repeated measures ANOVA showed that neither total protein nor albumin concentrations were affected by the independent variables (*p* > 0.05, data not shown). With regards to the control group, analytical values of TAC assessed by all four analytical methods did not change throughout the experimental period (*p* > 0.05), whereas significant differences appeared in the stressed group at the first sampling after DEX-treatment (day-7) for FRAP, and at the second sampling after DEX-treatment (day-14) for ABTS, FRSA, and TTL (Figure 1).

FRAP (Figure 1a) increased significantly at day 7 and 14 in the stressed group, in comparison to the control group (*p* < 0.001). On day 21, there was no longer a statistical difference, although the *p*-value was on the borderline (*p* = 0.057).

TAC, assessed by the ABTS^•+^ decolorization assay (Figure 1b), in the stressed group was significantly different only at day 14, compared to the control group, but at a low significance level (*p* < 0.05). With regards to the different sampling times in the stressed group, only the comparison between data obtained at day 7 and day 14 showed significantly different results (*p* < 0.01).

FRSA dropped significantly on day 14 (Figure 1c), reaching about 25% of the value of the control group (*p* < 0.001). On day 21, the values of the stressed group showed a remarkable recovery, differing neither from the value observed on day 0 in the same animals nor from the value of the control group at the same sampling time (*p* > 0.05).

TTL of the stressed group showed a significant reduction at day 14 and 21, compared to the control group (*p* < 0.001) (Figure 1d). The levels observed on day 21 in the stressed group, despite being statistically higher as compared to those observed on day 14 in the same group (*p* < 0.001), were still far from a full recovery, as shown by the comparison between TTL at day 0 and day 21 of the stressed group (*p* < 0.001).

## 4. Discussion

Four related methods were applied to assess the total antioxidant capacity (TAC) in serum samples from healthy and DEX-stressed hens. Although all assessed tests are designed to measure the total antioxidant status in biological samples, the obtained results suggest that the applied methods could lead to different conclusions for the same set of samples. First, FRAP, FRSA and TTL tests showed more intense and prolonged changes over time, due to the induced stress, as compared to those obtained by the ABTS test. Indeed, the ABTS assay showed a significant effect of DEX-treatment, but at the lowest significant level (*p* < 0.05) and only at day 14 from the beginning of the experiment. Second, the FRAP assay was the only test cable of showing a significant difference immediately after the DEX-treatment (day 7), while the other applied tests showed a slower ability to detect changes in the antioxidant status. Third, as a consequence of the induced stress, the values of FRAP and the ABTS assays increased, whereas in the FRSA and TTL assays, the values decreased. Fourth, at the end of the experimental period (day 21), the ABTS and FRSA values showed a complete recovery in the stressed group, whereas the TTL values were still statistically lower in the stressed group compared to the control group, and to the same group before the DEX-treatment. Furthermore, with regards to the FRAP test, although there was no significant difference between the control group and the stressed group on day 21, the values in the stressed animals were still statistically higher than those observed on day 0.

The interpretation of the changes of TAC in the biological samples, depends on the method used in detecting these changes and on the technology upon which it is based. Regarding the FRAP assay, its main contributors in human samples are uric acid, which represents about 60% of the final value, a-tocopherol, bilirubin and ascorbic acid [24], whereas it does not measure the SH-group containing antioxidants, such as glutathione (GSH) and albumin. This feature is considered by the authors to be an advantage for the evaluation of the antioxidant barrier in biological samples [24], since the method excludes endogenous substances of high molecular weight, such as albumin, from the evaluation. In the present experiment, in which a corticosteroid was applied to induce a reversible stressful condition [12,23], an enhanced uric acid level should be expected, as previously shown in chick [29], which could exceed 100% of the basal value in corticosterone-feeding animals [15]. Therefore, the increase in FRAP values in the present experiment could be mainly, if not exclusively, due to the enhancement in uric acid concentrations in the blood samples of the stressed animals.

In addition to the substances contributing to the FRAP value, the ABTS test also measures albumin, which represents about 50% of the final value in human samples, while the contribution of uric acid in the final ABTS value is lower than that in the FRAP assay [30]. In the present experiment, neither total protein nor albumin concentrations were affected by the induced stress. Therefore, the lower sensitivity of the ABTS assay in showing an imbalance of the antioxidant status compared to the FRAP assay could be explained by the different technology on which the two tests are based. TTL assay is designed to measure the occurrence of total thiol groups in serum or plasma, mainly represented by the sulfhydryl groups of albumin, l-cysteine, and homocysteine, which are considered for their pivotal role in the extracellular antioxidant defense system against oxidative stress [31]. It was reported that, under oxidative stress, the protein sulfhydryl (-SH) groups are lost [32], because the thiol groups can undergo oxidation to form disulfide bonds [33]. In the present experiment, TTL significantly decreased as a consequence of DEX-induced stress, a sign of occurred oxidation, as observed during the peripartum period, both in dairy cows and goats, in which a simultaneous increase of reactive oxygen metabolites was shown [34,35]. Although simple to perform, FRSA, assessed as DPPH reduction assay, is rarely used for TAC evaluation in the blood of living beings [36,37], mainly adopted for measuring the antioxidant content in food and plant extracts [38].

Nevertheless, DPPH assay was also used for TAC evaluation both of plasma and tissue samples in broiler chickens [39,40]. As stated by Kedare and Singh [38], DPPH reduction assay has a serious limitation in the assessment of the total antioxidant potential in biological samples. Indeed, since an alcoholic reaction medium is used in the analytical procedure, plasma proteins are precipitated before spectrophotometric reading. Therefore, their contribution to the evaluation of the total antioxidant activity is lost. Despite the reported limitations and according to the data of the present experiment, this method is useful for describing the induced stressful condition, probably measuring the lack of antioxidant substances of non-protein origin in samples, consumed to counteract the increased level of oxidants. The present data reinforce the idea that the TAC does not have an absolute meaning, but depends on the method employed for the evaluation. The observed differences in the total antioxidant values assessed by the different analytical methods could be explained by the discrepancies in the reactivity of various antioxidants, which contribute to the total antioxidant status with the respective indicators. Therefore, since there is no ideal method to assess the antioxidant potential in biological samples, the simultaneous use of different assays is strongly recommended to dutifully verify any changes in the total antioxidant barrier.

## 5. Conclusions

The observed differences in the total antioxidant values assessed by the different analytical methods could be explained by the different contribution in each assay of the various antioxidant substances present in the biological samples. Therefore, this study suggests that the evaluation of TAC should involve multiple assays, due to the different analytical technologies on which their assessments are based. The simultaneous use of several analytical assays should be considered for its key role in assessing the antioxidant potential in hen serum.
